# Rhetoric of psychological measurement theory and practice

**DOI:** 10.3389/fpsyg.2024.1374330

**Published:** 2024-04-18

**Authors:** Kathleen L. Slaney, Megan E. Graham, Ruby S. Dhillon, Richard E. Hohn

**Affiliations:** Department of Psychology, Simon Fraser University, Burnaby, BC, Canada

**Keywords:** psychological measurement, rhetoric, rhetoric of science, validation, metascience, methodological reform

## Abstract

Metascience scholars have long been concerned with tracking the use of rhetorical language in scientific discourse, oftentimes to analyze the legitimacy and validity of scientific claim-making. Psychology, however, has only recently become the explicit target of such metascientific scholarship, much of which has been in response to the recent crises surrounding replicability of quantitative research findings and questionable research practices. The focus of this paper is on the rhetoric of psychological measurement and validity scholarship, in both the theoretical and methodological and empirical literatures. We examine various discourse practices in published psychological measurement and validity literature, including: (a) clear instances of rhetoric (i.e., persuasion or performance); (b) common or rote expressions and tropes (e.g., perfunctory claims or declarations); (c) metaphors and other “literary” styles; and (d) ambiguous, confusing, or unjustifiable claims. The methodological approach we use is informed by a combination of conceptual analysis and exploratory grounded theory, the latter of which we used to identify relevant themes within the published psychological discourse. Examples of both constructive and useful or misleading and potentially harmful discourse practices will be given. Our objectives are both to contribute to the critical methodological literature on psychological measurement and connect metascience in psychology to broader interdisciplinary examinations of science discourse.

## Introduction

The theory and practice of psychological measurement has long been debated from numerous perspectives. Less represented in these topics, however, is the concern of how psychological researchers and measurement scholars *communicate* their findings and perspectives with respect to the construction, validation and use of measurement instruments in psychology. The focus of the present paper is, thus, on the *conceptual* arena of psychological measurement; that is, on the ways in which psychological researchers – both measurement and validity specialists and researchers using and reporting on psychological measurement tools – write about psychological measurement and validity, more generally.

First, we provide a brief overview of the rhetoric of science scholarship, including work examining the use of rhetoric in psychological research. We then summarize several different ways in which rhetoric appears in psychological measurement discourse. We describe several common forms of rhetoric and other styles of writing in psychological measurement and validity scholarship and provide examples from the broad theoretical psychological measurement and validity literatures. Our discussion is further supported by examples collected from a sample of recently published research articles from a larger study we have been conducting on rhetoric of psychological science (Slaney and Wu, 2021; Slaney et al., 2024).

## Rhetoric of science

We begin by drawing a distinction between *discourse* and *rhetoric* and between *discourse analysis* and *analysis of rhetoric*. Whereas discourse extends to all forms of speech, writing, and communication, rhetoric is one of many possible features of discourse in which the speaker (writer, or communicator) intends to frame the message in such a way as to persuade or, at least, privilege a specific interpretation of the content at hand. Understood in this way, discourse analysis can be generally construed as the analysis of some form of speech, writing, or communication. The analysis of rhetoric pertains to analysis of forms of rhetorical discourse or rhetoric within a given discourse. The persuasive aspects of science discourse have long been recognized in philosophy of science circles (Overington, 1977). Science and technology studies scholars have also been concerned with tracking scientific discourse, oftentimes to analyze the legitimacy and validity of scientific claim-making (e.g., Zerbe, 2007). A subset of such scholarship has been concerned with rhetoric both as a feature of scientific discourse practice and a potential form of knowledge itself (Gross, 2006). Whereas the former contributes to the larger domain of metascience (i.e., serves as a way of understanding science and scientists; Gross, 2006), the latter is more epistemic in orientation (i.e., serves as a “way of knowing” itself).

*Rhetoric of science* is a subfield of this scholarship and is broadly defined as “the application of the resources of the rhetorical tradition to the texts, tables, and visuals of the sciences” (Gross, 2008, p. 1). It specifically concerns the forms of argumentation and persuasion that appear in scientific writing, including on philosophical, theoretical, and empirical topics relevant to science generally and within specific research domains. According to Kurzman (1988), rhetoric of science is central to the drawing of logical inferences (theoretical, empirical, statistical) by scientists. Further, Gaonkar (1993) states the “general aim of the [rhetoric of science] project is to show that the discursive practices of science, both internal and external, contain an unavoidable rhetorical component” (p. 267) and that “science is rhetorical all the way” (p. 268). Importantly, this should not be taken to suggest that science is *nothing more than* argument and attempted persuasion but, rather, that studying the rhetorical function and form of scientific discourses “has something important to contribute to our understanding of how science develops” (Ceccarelli, 2001, p. 177).

It is important to note that *metascience* has been viewed by some critical scholars as insufficient for dealing with deep-rooted conceptual problems within psychological science (e.g., Slaney, 2021; Malick and Rehmann-Sutter, 2022). We agree that metascience might leave little room for the examination of rhetoric and other forms of psychological science discourse if narrowly conceived as a domain of scholarship concerned only with whether the dominant methodology and methods of the natural sciences are being properly applied. However, here we advocate for a broader conception of metascience construed broadly as “science about science” or “research about research” and not restricted to either the natural sciences or to critiques of limited or faulty applications of quantitative methods. Framed in this way, metascience captures critical examinations of science discourse, connecting it to philosophy of science and science and technology studies scholarship, including rhetoric of science studies.^1^

## Rhetoric of psychological science

Psychology has only relatively recently become the explicit target of metascience scholarship on a broader scale but most of this has been in response to recent crises surrounding replicability of quantitative research findings and questionable research practices (QRPs) within the discipline (e.g., John et al., 2012; Open Science Collaboration, 2012, 2015; Lindsay, 2015). Despite work identifying common problematic discourse practices in the discipline (e.g., overly simplistic language; unclear, misleading or inaccurate content; and logical errors; Smedslund, 1991, 2015; Slaney and Racine, 2011, 2013; Lilienfeld et al., 2015; Slaney, 2017; Uher, 2022a,b), few studies have directly addressed the relevance of rhetoric of science scholarship for analyzing psychological science discourse or even recognized that psychological research has been both the target and a tool of rhetorical analysis (Carlston, 1987; Nelson et al., 1987; Bazerman, 2003).

Most of the work explicitly examining rhetoric in psychology has been done either by theoretical psychologists or critical scholars from other disciplines (e.g., science communication scholars; philosophers of science). The rhetorical aspects of the psychological research report have been the subject of some of the work of scholars external to the discipline. Bazerman (1987) traced the history of the “codification” of published research in psychology from stylesheets and supplements in the journal *Psychological Bulletin* through the first three revisions of the *American Psychological Association (APA) Publication Manual* (American Psychological Association, 1974, 1983).^2^ Although the broad implementation of the *APA Publication Manual* facilitates communication and simplifies interpretation of research findings, Bazerman suggests the appearance of “epistemological neutrality” is “rhetorically naïve” and perpetuates a psychological research discourse that amounts to “incremental encyclopedism.” In other words, the rigid APA publication format appears on the surface to merely “gather and report the facts” toward a progressively more and more complete description of behavior (Bazerman, 1987, p. 258, p. 273). For example, methods and results sections have become particularly technical and perfunctory, functioning more to protect researchers from claims of methodological error than to support innovative theory (Bazerman, 1987; John, 1992). In conforming to the highly accessible, yet excessively constraining, structure of the APA publication format, researchers do their best to appear to “tell it like it is” while at the same time putting their “best foot forward,” both of which are clearly forms of rhetoric (i.e., attempted persuasion; Simons, 1993). Walsh and Billig (2014, p. 1682) asserted that the rhetorical style of the APA research report has become the “virtual *lingua franca*” of the discipline. Katzko (2002, p. 262) referred to it as an “institutionalized form of argumentation.”

Carlston (1987) emphasized that, while it is true that the psychological research discourse is a legitimate *target* of rhetorical analysis, psychological research may also be a *tool* of such analysis because psychologists “study processes and phenomena that are central to language, stories, persuasion and other topics of rhetoric and hermeneutics” (p. 145). He asserted that many of the theoretical constructs at play in psychological discourse (e.g., “schema,” “emotion,” “memory,” “motivation”) are not just labels for the phenomena under study but, rather, are “summarizations of theories, histories, issues and arguments” (p. 147). Essex and Smythe (1999) echoed this notion and added that the reification of psychological constructs (i.e., treating them as concrete or objectively real) understood in terms of statistical correlations between scores on psychological measures is reinforced by a positivist legacy in psychological measurement theory and practice.

Rhetoric in psychological research discourse has also been examined from within the discipline (e.g., Danziger, 1990, 1996; Abelson, 1995; Morawski, 1996; Rose, 2011). Two of the most pervasive practices are what discourse analysts call nominalization and passivization (Billig, 1994, 2011, 2013). Nominalization is the use of nouns to express what are actually actions (e.g., “perception” instead of “to perceive”) and passivization is researchers’ use of passive phrasing in describing their own research activities (e.g., “A measure was administered” instead of “We administered a measure”; “Scores were obtained” instead of “We used the following scoring rule to form composite scores”). Billig (2011, 2013) argued such writing styles reify (i.e., create “fictional things”) and “big up”^3^ theoretical constructs by making them appear more noteworthy or intellectually rigorous. Such rhetoric gives the *appearance* of greater technical precision and objectivity and “depopulates” the texts of research discourse (i.e., of the *people* involved in the research; Billig, 1994). The problem with this is that although such writing styles may create more succinct discourse, when used to describe human actions, the sentences they produce tend to convey *less* information (e.g., about who is doing the actions and to whom; how the phenomenon of interest is being operationalized) than sentences using active verbs. Consequently, such terms can give the appearance of precision; yet the writer’s meaning may remain inexplicit and ambiguous. Moreover, such writing styles reflect a prevalence of vague, abstract or unclear writing in psychological science (Billig, 2013; Kail, 2019).

Drawing from Billig’s work, the first author of the current work has examined the rhetoric of psychological constructs, arguing that the heavy use in psychological research reports of passive voice and nominals in place of verb clauses has contributed to the reification of psychological constructs and the widespread ambiguity concerning the intended meanings of specific psychological constructs, as well as of the meaning of the term “construct” itself (Slaney and Garcia, 2015; Slaney, 2017). We argued such rhetoric provides a partial explanation for the pervasive practice in psychological discourse of confusing psychological *constructs* with the *phenomena* such constructs are intended to *represent*. Put another way, rhetoric partially explains why theoretical concepts (i.e., terms, conceptual models, theories) created by researchers are often confused with the phenomena those concepts are meant to describe. Where there are such ambiguities surrounding the ontological status of psychological constructs (i.e., what they *are*), it remains unclear what it would mean to “measure,” “experimentally manipulate,” “assess,” “tap into,” “investigate” or “validate” one, all of which are practices central to psychological measurement theory and validation.

In other work, we identified two areas in addition to the rhetoric of constructs in psychological research discourse: the *rhetoric of crisis* and the *rhetoric of methodology* (Slaney and Wu, 2021). The rhetoric of crisis refers to the more recent attention given to the “replication crisis” and a host of QRPs in psychology. The rhetoric of methodology represents a broader set of discourse practices, including rhetoric surrounding psychological measurement. The “quantitative imperative” identified by Michell (2003), according to which psychological attributes are presumed to have inherent quantitative structure and are therefore measurable, is one example (Michell, 2003). Another example is the pervasive “language of variables” which replaced the language of the “stimulus–response” unit in the latter half of the twentieth century to accommodate the then growing practice of building theory through the ongoing establishment of correlations among psychological measurements (Danziger, 1996; Toomela, 2008). A third example is the common practice of psychological researchers reporting that the measures used in their studies are “reliable and valid,” often with no additional information or evidence about the psychometric properties of the measurement data from their studies (Weigert, 1970; Lilienfeld et al., 2015).

Additional critiques of conventional conceptions of and approaches to psychological measurement have identified other issues relevant to the present discussion. Tafreshi et al. (2016) argued that the quantitative imperative is one of several motivations for quantifying information in psychological research. Other motivations include the perceived need of ensuring objectivity, precision and rigor, reliance on statistical inference and adherence to both positivist and realist philosophies of science (Porter and Haggerty, 1997). In other work, the quantitative imperative has been addressed from a conceptual perspective, questioning the coherence of the very question of whether psychological attributes are measurable (see, for example, Maraun, 1998, 2021; Bennett and Hacker, 2022; Franz, 2022; Tafreshi, 2022; Tafreshi and Slaney, in press). Toomela (2008) argued that the implicit assumption that variables (i.e., data generated from the administration of psychological measures) directly represent the mental phenomena is based on faulty reasoning that there is a one-to-one correspondence between mental phenomena and behavior (i.e., measured variables). Lamiell (2013, p. 65) identified “statisticism” – the “virtually boundless trust of statistical concepts and methods to reveal” psychological laws – as fundamental way of thinking in contemporary psychological science. Uher (2022a,b) described several common conflations psychological and other social researchers make about measurement (e.g., data generation versus data analysis; quantity versus quality; measurement versus quantification). Bergner (2023) identified common scale construction practices based on confused concepts and flawed logic. It could be argued that these (and other) basic assumptions and practices of many psychological researchers are based more in a kind of perfunctory rhetoric than in scientific, theoretical or observational principles. Although they do not directly address the issue of rhetoric in psychological measurement literature, in a recent article, Flake and Fried (2020) identified an array of “questionable measurement practices” (QMPs), including everything from omissions of psychometric information to outright fraud and misrepresentation. One might contend that such “measurement flexibility,” when used to misrepresent or steer interpretations of study findings in a particular direction is an abuse of “epistemic authority” (John, 1992) and a form of rhetoric that should be made transparent.

## The current study: rhetoric and other discourse practices in psychological measurement and validity discourse

In the current work, we aim to dig a little deeper into the discourse practices of psychological researchers, specifically those related to psychological measurement. Our primary objective is to provide concrete examples of some common ways of writing about the uses and validation of psychological measurement tools and identify their potential rhetorical features. We draw from two different literatures, the first being the broad theoretical and methodological literature on psychological measurement and validation, the second a sample of recently published research articles. We explore both constructive and useful or misleading and harmful uses of the discourse practices.

## Method and results

### Sample

To explore the rhetoric and other discourse practices relevant to measurement and validation in the empirical psychological research literature, we reviewed a sample of recently published research reports from a larger project we have been conducting on rhetoric of psychological science (Slaney and Wu, 2021; Slaney et al., 2024). The initial sample (*N* = 40) combined two samples (each with 20 articles) from separate studies, one of which focused on the uses of cognitive and causal metaphors (Subsample 1), the other on discourse related to null hypothesis statistical testing procedures (Subsample 2; see Table 1). Articles in both samples were randomly selected from larger article databases representing issues published in 2021 in APA journals across a range of subject categories^4^ (~37 journals categorized as “Basic/experimental Psychology,” “Developmental Psychology” and “Neuroscience & Cognition” for Subsample 1 and 50+ journals categorized as “Basic/experimental,” “Clinical Psychology,” “Developmental,” “Forensic Psychology” and “Social Psychology & Social Processes” for Subsample 2). Due to overlap in the journals listed across the journal subject categories, we ensured that journals appeared only once. This created article populations of *N* = 561 and *N* = 266, respectively, for the first and second studies, from which we randomly sampled twenty articles from each. We included research reports on findings from quantitative data used in a single empirical study or on multiple studies reported in a single research report (i.e., by the same authors to address a set of hypotheses/research questions). We excluded editorials, commentaries, systematic reviews, non-English or strictly theoretical/methodological studies. One article from this sample was ultimately excluded, as the methods were deemed to be primarily qualitative with no use of quantitative measurement. Therefore, the final sample for the current study consisted of 39 articles.

**Table 1:** Journals represented in each subsample.

Sample	Journal
Subsample 1	*Neuropsychology*
	*Group Dynamics: Theory, Research and Practice*
	*Journal of Consulting and Clinical Psychology*
	*Psychological Trauma: Theory, Research, Practice, and Policy*
	*Journal of Experimental Psychology: Animal Learning and Cognition*
	*Experimental and Clinical Psychopharmacology*
	*Journal of Diversity in Higher Education*
	*Sport, Exercise, and Performance Psychology*
	*Psychology of Violence*
	*Emotion*
	*Journal of Abnormal Psychology*
	*Psychology, Public Policy, and Law*
	*Journal of Experimental Psychology: General*
	*Canadian Journal of Experimental Psychology/Revue canadienne de psychologie expérimentale*
	*American Journal of Orthopsychiatry*
	*Canadian Journal of Behavioural Science/Revue canadienne des sciences du comportement*
	*Journal of Family Psychology*
	*Psychological Assessment*
Subsample 2	*Canadian Journal of Experimental Psychology/Revue canadienne de psychologie expérimentale*
	*Psychology of Men & Masculinities*
	*Journal of Experimental Psychology: Human Perception and Performance*
	*Canadian Journal of Behavioural Science/Revue canadienne des sciences du comportement*
	*Neuropsychology*
	*Emotion*
	*Journal of Experimental Psychology: Applied*
	*Psychology of Aesthetics, Creativity, and the Arts*
	*Journal of Experimental Psychology: General*
	*Psychology and Aging*
	*Developmental Psychology*
	*Psychoanalytic Psychology*
	*Dreaming*
	*Experimental and Clinical Psychopharmacology*
	*Journal of Experimental Psychology: Learning, Memory, and Cognition*

### Procedure

Two research assistants independently reviewed and coded articles for a range of discourse practices including: (a) clear instances of rhetoric (i.e., persuasion or performance); (b) common or rote expressions and tropes (e.g., perfunctory claims or declarations); (c) metaphors and other “literary” styles; and (d) ambiguous, confusing, or unjustifiable claims. Coding categories were loosely defined *a priori*, though we left open the possibility of emergent themes.

Of the 39 articles, 20 were first reviewed and coded by both research assistants and the coding of the remaining 19 articles split between the two research assistants. Blocks of text were excerpted and then coded in terms of the categories described above. For those articles coded by both research assistants, overlapping excerpts were reconciled into a single entry in our textual database. We resolved discrepancies in coding through discussion with the entire research team and reflected finalized codes in the database. Though research assistants found multiple instances of a single code within a single article, the counts we report here of specific discourse practices capture the number of articles that contained at least one instance of a specific code. The final dataset was reviewed and vetted by the first author.

Before considering the results of this study, it is important to emphasize that our primary objective is not to make strong inferences strictly based on our sample about the prevalence of the discourse practices we have categorized herein. Rather, our main objective is to explore the conceptual landscape of validation and psychological measurement discourse practices – through *both* the theoretical and empirical literatures – to identify some of the ways in which psychological researchers use specific styles of writing to convey their understandings of measurement and validation tools, as well as the data generated from such tools. As such, the present study is better positioned as a conceptual analysis rather than as an empirical review of the theoretical and empirical psychological measurement and validation discourses at large. The results we present are meant to illuminate where such discourse practices are useful, benign or where they may be detrimental and potentially at odds with the intentions of psychological researchers.

### Results

#### Persuasive rhetoric of measurement

Michell (2003) argued the relevance and appropriateness of psychological measurement is almost universally *assumed* by psychological researchers. Although this does not constitute an obvious attempt to persuade, that very few psychological researchers question the feasibility of psychological measurement could be seen as a form of implicit persuasion that pervades both theoretical and empirical psychological research discourses. Of course, there are more explicit forms of rhetoric surrounding psychological measurement validation. The very objective of validation research is to provide compelling evidence that a measure or measurement data are valid in one or more of the many senses that exist of psychometric validity. Such research clearly plays an important role in persuading readers and consumers of research that a given measurement tool meaningfully quantifies the putative trait it was designed to measure or assess. In fact, it is now very common in empirical research reports to include evidence for justifying the use of the measures used in the study at hand.

The importance of providing persuasive evidence for measurement tools is also reflected in methodological standards and guidelines of the discipline. For example, the American Psychological Association (APA) Publication Manual (American Psychological Association, 2020) specifies an array of *journal article reporting standards* (JARS),^5^ including for reporting psychometric information concerning measurement data, the instruments used to generate these, and all other relevant psychometric information. Although clear reporting standards are essential within any scientific discipline, it is important to acknowledge the potential drawbacks Bazerman (1987) and others have identified that accompany overly rigid codification of research reports. Perfunctory reporting of psychometric information is a poor replacement for clear *demonstration* that the measures used and measurements generated in research studies are appropriate for study objectives.

In our article sample,^6^ we found examples of explicitly persuasive references to “important findings,” “substantial links,” “strong indicators,” and “robust” measures (e.g., models, effects, etc.), and “rich and informative” theoretical models. Some of these claims were not supported directly with empirical evidence and in some cases even accompanied *weak* empirical evidence, counter to the descriptions of “strong” or “robust” findings. We also found less direct appeals to the importance of study findings, such as references to the production of “useful” knowledge, “novel findings,” “advancing” knowledge in face of paucity of research or “gaps in the literature” and references to “confirming,” “reaffirming,” “reinforcing” expectations or findings from previously published research. Not surprisingly, most articles in our sample made as least one reference to “reliable” or “valid” measures or to the “reliability” or “validity” of the measures used in the study, over half of which (29 articles for “reliable”/“reliability” and 24 articles for “valid”/“validity”) either reported no direct evidence or vaguely gestured to previously published psychometric evidence. Examples of each of these kinds of explicitly persuasive forms of rhetoric are given in Table 2.

**Table 2:** Article sample results.

Sample article	Example
**Persuasive rhetoric of measurement**	
Explicitly persuasive forms of rhetoric	
Reported a “substantial link” between the independent and dependent variables where estimated effects were normatively small (i.e., r = 0.17 and d = 0.34).	Article 1
Explicit reference to the importance of “objective measures,” without elaboration of what constitutes objective in reference to the measure used.	Articles 6, 18, 33
Stated the measure used in the study “has undergone rigorous evaluation and been found to perform well relative to similar measures,” without reporting explicit psychometric evidence to justify.	Article 19
Described instrument used in study as the “gold standard” for the assessment of the phenomenon without elaboration of why this marker of excellence was provided.	Article 36
**Common or rote expressions and tropes**	
Vague gestures to previous research, validity, and reliability	
“Previous research has shown that…measures are more sensitive to [focal phenomenon].”	Article 2
“Previous research finds the [measure]has adequate test–retest reliability.”	Article 19
“Previous research has demonstrated the validity of [the measure].”	Articles 28, 37
Reported “reliability and validity” as a general property.	Articles 12, 13, 22, 27, 31
**Metaphors and other literary styles**	
Metaphors	
Measure was described “tap[ping]^*^ children’s ability to suppress a dominant response and undertake a subdominant response.”	Article 5
“The results revealed^*^ a significant three-way interaction between age group, condition, and perceived partner closeness.”	Article 26
References to “emerge” or “emerging” in relation to measured phenomena.	Articles 5, 12, 20, 21, 22, 27, 29
References to “detect” or “detection” in relation to measured phenomena.	Articles 1, 2, 6, 8, 17, 19, 21, 22, 25, 26, 28, 30, 32, 33, 35, 38
Use of “metaphorical story-telling” (Carlston, 1987).	Articles 16, 20
**Use of passive voice**	
“The Structured Clinical Interview for the DSM–IV, nonpatient edition … was administered^*^ to assess for Axis I DSM–IV disorders.”	Article 15
“Reward valuation ability^*^ was assessed…”	Article 18
**Misascribing actions or capacity**	
e.g., “the measure^*^ assessed” or “*items* access” as opposed to “We [the researchers] assessed … with the measure/items,”“this study^*^ conceptualized…” instead of “We conceptualized…”	
A growing literature has explored…” instead of “A growing number of researchers have explored…”	Articles 3, 4, and 12
**Confusing expressions, ambiguous, or unjustifiable claims**	
Construct validity	
“Such improvements in ADHD knowledge, use of behavioral strategies, and adaptive thinking skills, as measured by our study-specific measures, speak to their potential role as clinical change mechanisms, lending support to the construct validity of our design^*^”	Article 3
“[Cited authors] have provided evidence for the construct and criterion-related validity of this measure.”	Article 31
**Constructs**	
“As implicated in [cited study] meta-analysis, alliance is a *living*, ^*^ *evolving*, and *dynamic construct* that can be *perceived* and *reported* differently throughout the course of therapy.”	Article 1
Describe the construct of “functioning” as representing^*^ “a rather multifaceted construct, whose complexity may not have been captured by [the measure].”	Article 16
Described the relationship between the focal construct and other constructs as follows: “anxiety, depression, and posttraumatic stress disorder (PTSD) are constructs that display^*^ significant overlap with alexithymia.”	Article 18
Generativity is a distinct construct driven by^*^ the *underlying desire* to contribute to the community and future generations through one’s own legacy.”	Article 34
**Missing Information**	
“It is beyond the scope of this article to report on all of the behavioral outcomes that were assessed in the current study but, in addition to measures of subjective response…”	Article 19
**Hedging**	
“Various measurement approaches have been utilized in the field … Each of these measurement approaches has associated advantages as well as disadvantages and may capture distinct aspects of daily life.”	Article 27
**Other**	
Conflating ordinary and technical meanings of terms (e.g., reliable [*as in* dependable] measurement tools and measurements demonstrating high psychometric reliability).	Articles 1, 3, 5, 8, 17 and 30
Conflating aggregate statistical findings with individual-level causal claims (e.g., “Previous research has demonstrated the validity of this manipulation, showing, for example, that social exclusion makes individuals more aggressive … and reduces prosocial behavior,” and “Participants in the frustration condition further reported lower levels of satisfaction of the need for self-esteem”).	Article 28
**Confusing statements**	
“[Cited article] reported that the [measure] can be applied in a four dimensional or unidimensional structure to collect data with good reliability and validity.”	Article 13
“…the experimental design could detect the presence/absence of the [measure] effect moderately well, but likely does not reliably detect small changes in the [measure] effect across conditions. To reliably detect a 15 ms change in the [measure] effect at roughly 80% power, for example, we estimate would require 100 participants per group.”	Article 21

^*^Emphasis added.

#### Common or rote expressions and tropes

As with methodology discourse practices generally, there are some expressions and turns of phrase that have become prevalent in psychological researchers’ reporting of psychometric properties. As first illuminated by Weigert (1970), it is extremely common for psychological researchers to merely state that the measures used are “reliable and valid” or have “good,” “acceptable” or “sufficient” reliability and validity, often with no definitions of or distinction made between these concepts or evidence provided for the putative reliability or validity of the measurements or measurement instruments in question. The use of such rote expressions presents numerous problems, including that reliability and validity are quite different psychometric properties and, in the case of validity, bear on multiple different aspects of measures and measurements and uses thereof; that both may be assessed with different metrics (depending on the nature of the scale of measurement); and that reliability is required for validity but not vice versa. Another problem is that ordinary and technical senses of reliability become conflated when references are made to reliable and valid *measures* as opposed to of *measurements* (i.e., data): To state that a *measure* (i.e., the measurement instrument itself) is reliable (i.e., dependable, suitable) is quite a different claim than to state that *measurements* (i.e., scores or data from administering the measure) have strong psychometric reliability (i.e., a low ratio of error variance to observed variance of scores on a random variable). Another example of rote-like reporting on psychological measurement is the common practice of cursorily reporting only traditional aspects of validity (i.e., content, criterion-oriented [predictive and concurrent] and construct), which fails to reflect the seven decades of validity theory and methodology since Cronbach and Meehl’s seminal 1955 article (Cronbach and Meehl, 1955). In which validity was narrowly conceptualized in terms of these three broad types.

In our sample, phrases combining reliability and validity into a seemingly single psychometric property (i.e., “reliability and validity,” “reliable and valid”) did appear in the main body of some of the articles in our sample (see Table 2). The descriptor “good” was used often and to qualify everything from general reliability and validity or “psychometric/measurement properties” to specific kinds of validity (e.g., “model fit,” “convergent”) or reliability (e.g., “test–retest,” “internal reliability,” “stability,” “agreement”). There appears to be at least some degree of rhetorical motivation for these appeals to “goodness,” given that typically little elaboration was provided. Such underspecified claims appear to rhetorically stand in for any direct evidence of the psychometric properties of the measure being used to generate data for the study.

#### Metaphors and other literary styles

The use of metaphors in scientific discourse is hardly rare and there have been many celebrated cases in the physical and life sciences (e.g., Bohr’s “planetary” model of the hydrogen atom; evolutionary “tree” of life; DNA as a “twisted ladder”). Psychological measurement discourse also contains some commonly used metaphors, such a “tapping” “probing,” and “emerging” in reference to putative fundamental factors or “constructs” said to “underlie” an observed correlation matrix of a set of item or subscale scores. Item-level scores are framed as “indicators” of “latent” factors, the latter of which are sometimes described as “driving” observed relations among item-level or subscale scores. Other common literary styles include the use of passive voice (e.g., “the measure *was administered* to…”; “…*was assessed by…*”) and nominals in place of verb clauses (e.g., “…measure the *construct of* extraversion”) of the kind Billig (2011) has identified. Both the uses of passive voice and nominalization of actions and activities of persons into traits presumed be “tapped” or “probed” by psychological measures constitute examples of depopulating texts, whereby the specific researchers making and acting upon decisions about the measurement tools used in their research become obscured. Such discourse styles serve a “rhetoric of scientificity” (Bourdieu, 1975) which is intended to give the impression that the research was conducted rigorously and objectively and, therefore, the findings can be trusted.

In our sample, each of the articles contained metaphors of one kind or another. The most common terms were “tap” (or “tapping”) in relation to the phenomenon putatively measured or assessed and “reveal” (or “revealing”) in reference to data or findings. We found that the terms “tap” and “reveal” were used to convey that measurement data had unveiled an underlying or latent realm. Across the sample, other common metaphors were “emerge/emerging” and “detect/detectable/detection.” More unique metaphor use was exemplified by “metaphorical storytelling” (Carlston, 1987), in which a concept or phenomenon is elaborated through a narrative style that relies on the use of metaphors. Examples of the use of these terms and discourse styles in our article sample are listed in Table 2.

We also found that the use of passive voice was ubiquitous in our article sample, appearing multiple times in every article (e.g., “was evaluated,” “was assessed,” “were measured,” “were observed,” “were obtained,” etc.). It was also common, for example, to see such references to the administration of tests such as: “The Structured Clinical Interview for the DSM–IV, nonpatient edition … *was administered* to assess for Axis I DSM–IV disorders” (Article 15; emphasis added). This example is particularly noteworthy as the assessment tool in question is not a survey or trait measure, but a clinical interview, something that is inherently grounded in human interaction. To remove the interviewer from the “administration” of this test is indicative of the rhetoric of scientificity mentioned above.

In our sample, authors’ use of nominals in place of verbs, as with the use of passive voice, was encountered in every article. This is not surprising, as it is virtually impossible to write efficiently without simplifying at least some verbal clauses with nominals (e.g., “perception” instead of “X perceived Y”), as Billig and discourse scholars have acknowledged. It has become so commonplace in social science writing that it is almost unnatural to describe human actions and capacities in verbal clauses.

Although not a literary device *per se*, it has become common in psychological discourse for writers to inappropriately ascribe to the subject of a sentence an action or capacity which could not, on logical grounds, be attributed to that subject (see examples in Table 2). Although such misattributions have become more common in contemporary discourse and often do not create too much confusion about what is being stated, they do contribute to the textual depopulating that Billig has identified as having a rhetorical aim.

#### Confusing expressions, ambiguous, or unjustifiable claims

All forms of discourse at times contain unclear or confusing expressions; psychological scientific discourse is no exception. Although encountering the occasional ambiguous claim does not always create problems, science does not thrive in the face of pervasive ambiguity, and certainly not in unjustifiable statements. The discourse surrounding psychological “constructs” is one area where confusion, ambiguity and, in some cases, unjustifiable claims are commonly encountered.

Discussion of constructs pervades psychological research across theoretical, methodological and empirical domains. Yet, nowhere is there more ambiguity in the psychological measurement and validity discourse then with the “ever-evasive” construct concept (Slaney, 2017). Not only is the ontology of psychological constructs fuzzy, it is often difficult to discern what relationship constructs have to putative psychological “traits” and “mechanisms” (“qualities,” “properties,” “inferred entities,” “processes,” etc.); factors or “latent variables”; or with theoretical concepts, operational definitions, theories, theoretical statements, models or hypotheses (Maraun and Gabriel, 2013; Slaney, 2017). That is, constructs have been variously and confusingly characterized as *concepts* (e.g., theoretical constructs, hypotheses, models, theories), *objects of inquiry* (i.e., real but unobservable or only indirectly measurable theoretical *entities*, or features thereof) and, more generally, as the particular domain under study (e.g., “executive functioning,” “prosociality,” “attachment”). In fact, that psychological characteristics of persons are referred to as “traits,” “mechanisms” and “processes” (and other such objectivist terminology) could be viewed as a form of rhetoric in presuming psychological attributes are just like physical traits, except that they are psychological in nature.

Although ambiguity is not itself an explicit form of rhetoric, if let unexamined it can carry rhetorical weight. For instance, in allowing constructs to be ontologically “fluid,” some claims by researchers might appear stronger on the face of it than they really are. For example, Colman (2006, p. 359) defines a (hypothetical) construct as “a conjectured entity, process, or event that is not observed directly but is assumed to explain an observable phenomenon.” While this all sounds fine on the surface, it is unclear what it means for an “entity, process or event” to “explain” observable phenomenon. Although it has the ring of a precise scientific statement concerning the causal origins of the phenomenon under study, how the presence of causal structures and mechanisms could possibly be picked up by aggregate measurements is left unclear, at best. Similar ambiguities concerning the relationship between psychological constructs, observability and knowledge are prevalent in the discourse, as well as with other measurement-related concepts (e.g., “factor,” “variable,” “latent,” “uni/multidimensional”; see, e.g., Green et al., 1977; Maraun and Gabriel, 2013; Slaney, 2017). As noted by Flake and Fried (2020), such “unjustified measurement flexibility” compromises the extent to which sound evidence about the measures used in a study can be provided which, in turn, casts doubt on the study findings overall.

In our sample, approximately half the articles referred to either of the terms “construct” or “construct validity.” Construct validity was often claimed without direct appeal to psychometric evidence. For example, in some instances construct validity was presumed to be established through the common practice of simply invoking a previous single study. In one article, it was stated that “[s]uch improvements in ADHD knowledge, use of behavioral strategies, and adaptive thinking skills, as measured by our study-specific measures, speak to their potential role as clinical change mechanisms, lending support to the construct validity of our *design*” (Article 3; emphasis added). The references to both “clinical change mechanisms” and construct validity are vague, leaving unclear what is meant by the terms themselves, what the “construct” that has been validated is and how the results evidence the putative validity of said construct.

In terms of constructs themselves, authors from our sample referred to these without providing much if any indication of the specific natures of the constructs at hand. Several examples are listed in Table 2. Taking these examples together, it is difficult to determine the nature of psychological constructs such that they can be “driven by underlying” emotional states and considered to be “living” and “evolving,” but also to “represent” putative traits (attributes, etc.) and “display” relationships with other constructs.

We found other confusing or ambiguous forms of writing in our sample. These include reference to missing information and hedging. Additional examples include conflating ordinary and technical meanings of psychological concepts as well as conflating aggregate statistical findings with individual-level causal claims. We also found a small number of completely unclear or confusing statements. Examples of each of these kinds of confusing and/or ambiguous claims can be found in Table 2.

## Discussion

### What’s the problem with a little rhetoric?

#### Constructive versus destructive rhetoric

It is important to note that rhetoric of science scholars are not united in how they frame rhetoric in science discourse or whether they view it as useful and essential, harmful and misleading, or inevitable or avoidable. Haack (2007, pp. 217–223) draws an important distinction between “reasonable” and “radical” rhetoric of science and between “modes of communication that promote the epistemologically desirable correlation, and those that impede it.” She contrasts between two very different scenarios, one in which a scientific claim is accepted because clear and strong evidence is clearly communicated and the other in which a scientific claim comes to be accepted in the *absence* of good evidence because it is promoted by means of “emotive language, snazzy metaphors,… glossy photographs, melodramatic press conferences, etc.” (p. 223). Whereas Haack describes the first scenario as legitimately persuasive, she views the second as “strictly rhetorical.” Simons (1993) echoes something similar, noting that rhetorical argumentation does not necessarily make for bad argumentation; however, the slope from rhetoric to fraud may be slippery (Simons, 1993). More optimistically, Carlston (1987) characterizes an intertwining relationship between rhetoric and empirical science, wherein “empirical efforts complement but do not replace rhetorical practices, and rhetorical analysis illuminates but does not invalidate empirical pursuits,” and both are legitimate tools for accumulating “useful understandings and knowledge” (p. 156).

For example, on the use of scientific metaphors as one potential rhetorical strategy, Haack (2007) concedes that although they “oil the wheels of communication” and can be a source of new and important avenues of inquiry, “their worth…depends on the fruitfulness of the intellectual territory to which these avenues lead” (p. 227). Further, Haack notes, a given scientific metaphor may lead scientists in different directions, some better, some worse. As Nagel (1961; as cited in Carlston, 1987) warned over six decades ago, the use of scientific metaphors can be detrimental if the limits of their uses are not properly acknowledged and attended to.

It is fair to ask why scientists would not genuinely wish to persuade readers and consumers to accept research findings they believe are based on strong scientific practice. We agree with Haack that it would be quite counter-intuitive for psychological or any other researchers to avoid making persuasive claims that their research findings are both valid and important. At the same time, it is not always fully clear or agreed upon as to what constitutes “strong” or “good” evidence. Simply claiming strong or good evidence is questionable rhetoric. Moreover, there is no necessary connection between radical (poor) rhetoric and bad (weak) evidence: One can use radical rhetoric in reference to valid and strong evidence and reasonable rhetoric in reference to poor evidence.^7^ On the basis of the current sample of psychological research reports, we see that although some uses of rhetorical writing are relatively harmless (e.g., some nominalization, especially when its use is explicitly justified as descriptive efficiency) or even useful (e.g., metaphorical “story-telling” to clarify a concept), others create ambiguity, at least, and outright confusion, at worst. For example, sometimes using “variable,” “factor,” “construct,” etc. interchangeably is harmless, as the intended meanings of these terms in some contexts need not be precise (e.g., in highly general references to the phenomenon under study); however, in other instances, conflating these terms can be truly confusing, such as when constructs are portrayed as theoretical (explanatory) models and *at the same time* the putative trait measured by a given instrument. Clearly a construct cannot both be a theory and that which is the subject of the theory. Moreover, reifying aspects of psychological functioning through nominalization and other styles of discourse (e.g., “trait” terminology) can also affirm naïve naturalist and realist views on the nature of psychological reality, thus obscuring important conceptual connections between ordinary and scientific senses of psychological concepts (Danziger, 1990; Brock, 2015; Slaney, 2017; Tafreshi, 2022; Tafreshi and Slaney, in press).

#### Why is studying rhetoric and other discourse practices in psychological measurement scholarship important?

Of course, the answer to this question depends on who you ask, as even rhetoricians are divided on the question of where rhetorical analysis fits within the grand scheme of science (Simons, 1993). As noted at the beginning of the paper, we view examining rhetorical and other discourse practices as an important part of metascience, a primary aim of which is to improve science through better understanding *of* science (Ceccarelli, 2001), or of a given discipline or area of study (Overington, 1977) as it evolves within current social contexts. As such, it constitutes a part of recent movements within the discipline to acknowledge and address fundamental problems with psychological research (e.g., replication crisis; fraud; identification of QRPs, QMPs, etc.) and, in so doing, improve psychological science (e.g., Society for the Improvement of Psychological Science [SIPS]).^8^ We emphasize psychological measurement discourse not because it is unique in involving rhetorical features but because psychological measurement – even if not always explicitly acknowledged – provides the foundation for psychological research methods, more broadly. That is, a prevalence of questionable *measurement* practices “pose a serious threat to cumulative psychological science” and, yet, have received much less scrutiny and attention than failures of replication and other QRPs (Flake and Fried, 2020, p. 457), neither of which can be fully understood in the face of potentially widespread invalidity of the psychological measurement tools that generate the data which are the inputs for other psychological research methods.

It is also important to acknowledge that rhetoric and other discourse practices that might misrepresent the phenomena under study or otherwise create ambiguity or confusion occur neither in isolation nor in a vacuum. Most psychological research reports, including those in our sample, have been subject to peer and editorial review prior to publication.^9^ Yet, problematic discourse practices, such as those we have identified, manage to make it past the peer-review and editorial filters. This signals that the use of confusing or unclear language (rhetorical or otherwise) in psychological research discourse is a systemic problem, not to be blamed just on individual researchers. As with other QRPs that threaten the integrity of psychological research, a response is needed to address the *questionable discourse practices* in psychology that have been illuminated here and elsewhere. How researchers frame their theoretical positions, methods choices, the data that arises from their implementation, and the interpretations they make of findings should be, we argue, an essential part of the discussion about QMPs and QRPs. The upside is that illuminating the detrimental effects of such practices can, if taken seriously, be rectified by broad implementation of training in such areas as philosophy of science, metatheory, and scientific writing for psychology (Billig, 2013; Slaney, 2017; Kail, 2019; Uher, 2023). We believe that exposing pervasive hidden assumptions researchers take into their research can influence how reflective researchers (and, by extension, the discipline) will be regarding the relevant subject matters they are concerned with. We see the current work, and that of other critical methods scholars, as making important contributions to current discussions about methodological crisis and reform.

## Ethics statement

Ethical review and approval was not required for the study on human participants in accordance with the local legislation and institutional requirements. Written informed consent was not required in accordance with the national legislation and the institutional requirements.

## Author contributions

KS: Validation, Formal Analysis, Writing – review & editing, Writing – original draft, Supervision, Resources, Project administration, Methodology, Investigation, Data curation, Conceptualization. MG: Validation, Writing – review & editing, Writing – original draft, Supervision, Investigation, Formal Analysis, Data curation. RD: Writing – review & editing, Writing – original draft, Investigation, Data curation. RH: Writing – review & editing, Writing – original draft.

## Footnotes

^1^Uher (2023) uses “metatheory” to capture the philosophical and theoretical assumptions researchers hold about the phenomena they study. In the current work, because we focus on a set discourse *practices* within psychological science, we believe “metascience” better captures the kind of inquiry we are engaged in.

^2^Four revisions have since been published, in 1994, 2001, 2009, and 2019, respectively.

^3^Smedslund’s recent critique of “neuro-ornamentation” – the attempt to strengthen the impact of psychological study findings by inserting references to neuroscience – is another potent example of psychological researchers trying to “big up” the scientific relevance of their research (Smedslund, 2020).

^4^See https://www.apa.org/pubs/journals/browse?query=subject:Basic+%2f+Experimental+Psychology&type=journal

^5^The JARS guidelines largely reflect those published in 2008 by the APA Publications and Communications Board Working Group on Journal Reporting Standards (APA Publications and Communications Board Working Group on Journal Article Reporting Standards, 2008), which were updated in 2018 (Appelbaum et al., 2018).

^6^Because we treated the text from our sample of articles as a qualitative source of data, we have indicated article numbers rather than formal citations in the results described, including directly excerpted text. Citations will be made available upon requests made to the first author.

^7^We thank an anonymous reviewer for highlighting this.

^8^https://improvingpsych.org/mission/

^9^We thank an anonymous reviewer for raising this point.
